# Spatiotemporal variations, assembly processes, and co-occurrence patterns of particle-attached and free-living bacteria in a large drinking water reservoir in China

**DOI:** 10.3389/fmicb.2022.1056147

**Published:** 2023-01-19

**Authors:** Bobing Yu, Guijuan Xie, Zhen Shen, Keqiang Shao, Xiangming Tang

**Affiliations:** ^1^State Key Laboratory of Lake Science and Environment, Nanjing Institute of Geography and Limnology, Chinese Academy of Sciences, Nanjing, China; ^2^College of Resources and Environment, University of Chinese Academy of Sciences, Beijing, China; ^3^College of Biology and Pharmaceutical Engineering, West Anhui University, Lu’an, China

**Keywords:** particle-attached bacteria, free-living bacteria, assembly process, co-occurrence pattern, Lake Tianmu, spatiotemporal variations

## Abstract

Particle-attached (PA) and free-living (FL) bacterial communities are sensitive to pollutant concentrations and play an essential role in biogeochemical processes and water quality maintenance in aquatic ecosystems. However, the spatiotemporal variations, assembly processes, co-occurrence patterns, and environmental interactions of PA and FL bacteria in drinking water reservoirs remain as yet unexplored. To bridge this gap, we collected samples from 10 sites across four seasons in Lake Tianmu, a large drinking water reservoir in China. Analysis of 16S rRNA gene libraries demonstrated spatiotemporal variations in bacterial diversity and identified differences in bacterial community composition (BCC) between PA and FL lifestyles. Capacity for nitrogen respiration, nitrogen fixation, and nitrate denitrification was enriched in the PA lifestyle, while photosynthesis, methylotrophy, and methanol oxidation were enriched in the FL lifestyle. Deterministic processes, including interspecies interactions and environmental filtration, dominated the assembly of both PA and FL bacterial communities. The influence of environmental filtration on the FL community was stronger than that on the PA community, indicating that bacteria in the FL lifestyle were more sensitive to environmental variation. Co-occurrence patterns and keystone taxa differed between PA and FL lifestyles. The ecological functions of keystone taxa in the PA lifestyle were associated with the supply and recycling of nutrients, while those in FL were associated with the degradation of complex pollutants. PA communities were more stable than FL communities in the face of changing environmental conditions. Nutrients (e.g., TDN and NO_3_^–^) and abiotic and biotic factors (e.g., WT and Chl-*a*) exerted positive and negative effects, respectively, on the co-occurrence networks of both lifestyles. These results improve our understanding of assembly processes, co-occurrence patterns, and environmental interactions within PA and FL communities in a drinking water reservoir.

## Introduction

Reservoirs make primary contributions to essential ecosystem services such as the maintenance of biodiversity and providing sustainable water supplies for agricultural irrigation, human consumption, and renewable energy regeneration (Xie et al., 2021). Compared to natural rivers, artificial reservoirs usually have longer residence time, leading to a more rapid accumulation of pollutants and potential loss of ecological functions (Yue et al., 2021). Bacteria are extremely sensitive to pollutant concentrations in water (Yue et al., 2021) and play an essential part in biogeochemical processes and water quality maintenance in reservoir ecosystems (Zhang et al., 2015). Therefore, understanding the bacterial community composition (BCC) and spatiotemporal variations of these communities provides important insights into conservation and monitoring efforts, management decisions, and long-term planning of resource use (Roeske et al., 2012; Zhang et al., 2013).

For freshwater ecosystems, the local dynamics of microbiomes are attributed to microbial community assembly mechanisms controlling biodiversity patterns at both community and function levels (Ning et al., 2019). Community assembly has classically been classified as a deterministic process based on the niche theory, and the stochastic process based on the neutral theory, which jointly determines microbial biogeography. Understanding and elucidating the assembly processes of bacterial communities are the holy grail of microbial ecology (Liao et al., 2016; Tang et al., 2020; Zhang et al., 2022), from which predictions of future community composition and biogeochemical function in response to changing environmental conditions may be possible (Stegen et al., 2015). Co-occurrence is a key ecological attribute, reflecting niche processes that promote diversity and coexistence in biotic communities. Analysis of the underlying interactions between complex assemblages of microbes (e.g., predation, competition, inhibition, and facilitation) can reveal potential functions and ecological niches within these communities (Chaffron et al., 2010). Thus, exploring assembly processes and co-occurrence patterns can potentially contribute to the characterization of the biogeographical, functional, and ecological interrelationships of microorganisms.

Aquatic bacterial communities can be classified as either particle-attached (PA) or free-living (FL) depending on their functional lifestyle (Grossart, 2010). Ecological studies of the relationships between these two bacterial groups have focused mainly on communities found in marine systems (Lapoussiere et al., 2011; Yuan et al., 2021) and in both deep (Parveen et al., 2011; Roesel et al., 2012) and shallow lakes (Tang et al., 2015; Zhao et al., 2017a,b; Xu et al., 2018). In these studies, the bacterial diversity of PA was frequently found to be higher than that of FL bacteria (Crespo et al., 2013; Ortega-Retuerta et al., 2013; Zhao et al., 2017b). The structure of these two community types tends to vary across diverse ecosystems (Tang et al., 2015; Xu et al., 2018). For example, PA and FL lifestyles differed significantly in BCC in Lake Taihu (Zhao et al., 2017b) and in some open sea communities (Ortega-Retuerta et al., 2013), while being nearly identical in some rivers (Ortega-Retuerta et al., 2013). In these examples, the assembly processes associated with these two lifestyles were identified as different in Lake Taihu (Zhao et al., 2017b) and in the South China Sea (Yuan et al., 2021), and thus seem to be reflected in the differing BCC of each.

Co-occurrence patterns between PA and FL lifestyles have also been widely studied (Nemergut et al., 2013; Yang et al., 2017; Xu et al., 2018; Liu J. M. et al., 2022; Shen et al., 2022) and have yielded valuable findings. For example, Liu J. M. et al. (2022) observed that PA communities exhibited more complicated co-occurrence patterns than FL communities in coastal systems with high anthropogenic perturbations, while Yang et al. (2017) reported that FL networks contained more nodes and edges than PA networks during a *Microcystis* bloom. Furthermore, Shen et al. (2022) noted that PA networks were more stable than those of FL bacteria in shallow, eutrophic Lake Taihu, while Xu et al. (2018) demonstrated that bacterial communities of PA and FL networks are associated with different environmental conditions.

Despite the accumulating knowledge of the differences between PA and FL communities, little attention has been directed toward the effects of environmental conditions on the co-occurrence patterns of these two lifestyles in drinking water reservoirs. To bridge this knowledge gap, we collected 80 samples (40 PA samples and 40 FL samples) at 10 sites across four seasons in Lake Tianmu, a drinking water reservoir in east-central China. BCCs were determined by Illumina sequencing of sample libraries. Approaches based on the null model and analysis of co-occurrence were used to determine community assembly processes and disentangle factors contributing to co-occurrence patterns. This study tested the following hypotheses: (1) spatiotemporal variations and differences between BCCs exist in PA and FL bacterial communities; (2) deterministic processes dominate the assembly of PA and FL bacterial communities; and (3) disparities in co-occurrence patterns and interactions between species and environmental parameters result in differing stability and ecological functions in PA and FL bacterial communities.

## Materials and methods

### Study area and sample collection

Lake Tianmu (water area: 12 km^2^; mean depth: 7 m), located in Jiangsu Province, China, at present provides potable water for 790,000 residents of Liyang in addition to supplying water to local industries (Zhou et al., 2021). Approximately 80-90% of the volume of water in Lake Tianmuhu comes from the Zhongtian River in the south and the Pingqiao River in the south-east. Water from the lake flows eventually into Lake Taihu, the third-largest freshwater lake in China. Water samples were collected at 10 sites in Lake Tianmu during each of the four seasons: 14 April 2020 (spring), 14 July 2020 (summer), 14 October 2020 (autumn), and 14 January 2021 (winter) (Supplementary Figure 1).

For each site, 250 ml of water (top 0.5 m) was filtered sequentially through 5 μm and 0.22 μm pore-size membranes of polycarbonate (Millipore). Bacterial biomass captured on the 5 μm filters was considered PA bacteria, whereas those on the 0.22 μm filters were considered FL bacteria. All filters were preserved at −80°C until further analysis. The filtrate was captured, refrigerated at 4°C, and used for chemical analysis.

### Measurements of environmental parameters

Water temperature (WT), dissolved oxygen (DO), and pH were quantified *in situ* with a YSI EXO2 sonde (Yellow Spring, OH). The concentration of nitrate (NO_3_-N), total nitrogen (TN), total dissolved phosphorous (TDP), orthophosphate (PO_4_^3–^), total phosphorus (TP), chlorophyll-a (Chl-a) total dissolved nitrogen (TDN), ammonium (NH_4_^+^-N), and dissolved organic carbon (DOC) were quantified based upon the standard approaches (Jin and Tu, 1990). The Kruskal–Wallis test was used to test for significant differences in these parameters between seasons. The values of physicochemical parameters are exhibited in Supplementary Figure 2.

### Bacterial diversity and community composition

DNA extraction was conducted using FastDNA^®^ SPIN Kit for Soil (MP Biomedicals, Solon, OH, USA) following the manufacturer’s instructions. The V3–V4 regions of the bacterial 16S rRNA gene were amplified using 338F/806R primers (Fadrosh et al., 2014). PCR reactions were performed as previously described (Xie et al., 2021). The amplicon pools were purified using AMPure XP beads. Validated PCR products were sequenced on the Illumina MiSeq platform producing 2 × 300 bp PE reads.

Bioinformatic analysis including quality control and classification and clustering of operational taxonomic units (OTUs, >97% similarity) was performed using CLC Genomics Workbench 20.0 (Christensen, 2018). To minimize the random sequencing errors, low abundance OTUs (<10 reads) were filtered out. Taxonomy was assigned at the 80% confidence level by comparing reads to the SILVA database (Quast et al., 2012).

To normalize sequencing depth, 8,463 sequences were selected randomly from each sample. This number was equal to the number of quality sequences obtained from the sample producing the fewest number of reads. Estimates of α-diversity (Shannon and Chao 1 indices) were quantified using the R package “vegan” (v4.2.1). The Kruskal–Wallis test was used to test for differences in α-diversity between seasons and types (PA vs. FL). Non-metric multidimensional scaling (NMDS) based on Bray–Curtis distance was used to calculate bacterial β-diversity. Analysis of similarity (ANOSIM) was used to test for differences in BCC between lifestyles (BCC_PA_ vs. BCC_FL_) and seasons.

### Functional annotation of bacterial communities

Bacterial functional composition (BFC) was annotated according to the FAPROTAX_1.1 (Louca et al., 2016, 2018) database. Differences in BFC between the PA and FL fractions were visualized with STAMP v2.0.4 (Parks et al., 2014).

### Environmental and spatial factors associated with BCC

Redundancy analysis (RDA) using the R package “vegan” was used to identify environmental parameters or combinations thereof correlated to BCCs. In subsequent analyses, OTU data were Hellinger-transformed, and environmental (explanatory) parameters were normalized to remove differing measurement scales. Significant environmental parameters were then identified using a forward selection procedure employing a Mantel test with 999 permutations. A variance inflation factor (VIF) was calculated for each significant parameter, and parameters with VIF values above 10 were eliminated. Subsequently, the contribution of significant environmental parameters or combinations thereof was calculated using the package “rdacca.hp” (Lai et al., 2022). Mantel tests and partial Mantel tests using 999 permutations and computed within the “vegan” and “linkET” packages in R were used to calculate the Spearman correlation among BCC (Bray–Curtis distance), geographical distance (Euclidean distance), and environmental parameters (Euclidean distance) (Tang et al., 2021). Principal coordinates of neighbor matrices (PCNM) using Hellinger-transformed OTU data and a matrix of geographical distance were applied to assess the relative contributions of spatial factors (Borcard and Legendre, 2002; Tang et al., 2021). From the results of the RDA and PCNM analyses, the contribution of spatial factors and environmental parameters in the construction of bacterial communities was then calculated using the variation partitioning approach (VPA).

### Assembly processes of bacterial community

To estimate the average stochasticity of PA and FL communities, the modified stochasticity ratio (MST), which is a special form of Normalized Stochasticity Ratio (NST), was calculated using the R code based upon Jaccard (incidence-based) dissimilarity metrics and taxonomic β-diversity metrics. MST has a value range of 0 to 1, with a value of 0.5 serving as a threshold between assembly process types. Stochastic assembly processes (chance colonization, demographic randomness, and ecological drift) predominate in communities with an MST distributed above 0.5, while deterministic assembly processes (environmental filtration and interspecific interaction) predominate in communities with an MST distributed below 0.5 (Ning et al., 2019).

### Construction and analysis of co-occurrence networks

Co-occurrence patterns of PA and FL lifestyles were constructed based on the network theory. These patterns were analyzed to gain a deeper understanding of interactions among bacterial taxa on assembly processes. To simplify the dataset and improve the credibility of network analysis, only those OTUs with a relative abundance of >0.08% were selected for analysis. In addition, we included environmental factors in the network to assess the ecological associations between these factors and community species. Nodes within the network represent environmental parameters or OTUs. Edges linking two nodes represent negative or positive connections. The significance matrix (*p*-values) and correlation matrix (*R*-values) were calculated using Spearman’s rank correlations using the R package “psych.” Spearman correlation coefficients | *r*| > 0.80 with *p* ≤ 0.01 were selected for use in species–species and species–environment network analyses. Network topological attributes (refer to Supplementary Table 1), including graph density (GD), average degree (AD), average clustering coefficient (AvgCC), modularity, average path length (APL), and 100 random networks, were quantified using “igraph” package in R (Deng et al., 2012). The stability of networks was estimated *via* robustness (Deng et al., 2012).

Two parameters can be calculated for the resulting networks: *P*_*i*_ (among-module connectivity) and *Z*_*i*_ (within-module connectivity) (Liu S. W. et al., 2022). The values of *P*_*i*_ and *Z*_*i*_ were calculated using the “igraph” package in R. OTUs within a network can be grouped into one of four topological classes based on calculated values of *P*_*i*_ and *Z*_*i*_: network hubs (*P_*i*_* > 0.62, *Z*_*i*_ > 2.5), connectors (*P*_*i*_ > 0.62, *Z*_*i*_ ≤ 2.5), module hubs (*P*_*i*_ ≤ 0.62, *Z*_*i*_ > 2.5), and peripheral (*P*_*i*_ ≤ 0.62, *Z*_*i*_ ≤ 2.5) (Liu S. W. et al., 2022). Scatterplots of *P*_*i*_–*Z*_*i*_ were visualized using the “ggplot2” package. Co-occurrence networks were implemented in Cytoscape (v3.6.0) and Gephi (v0.9.1).

## Results

### Diversity, community, and functional composition of the PA and FL lifestyles

**FIGURE 1:**
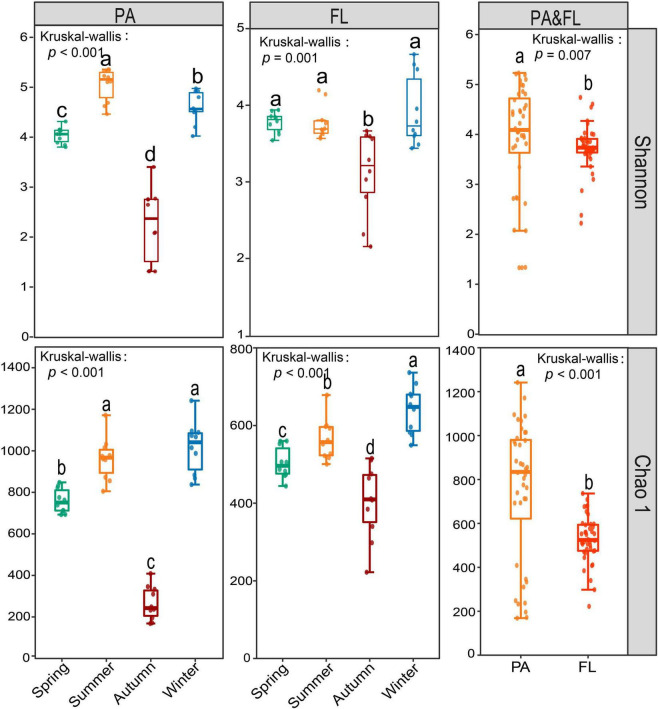
The comparison of α-diversity indices of the particle-attached (PA) and free-living (FL) lifestyles in Lake Tianmu. Kruskal–Wallis test was used to test the significance level of the differences. Different lowercase letters above each boxplot indicate significant differences (*p* < 0.05) among seasons and between fractions.

From all these 80 samples, we generated a total of 3,017,192 high-quality reads that were divided across 2,831 OTUs. A high proportion of OTUs was shared within the PA and FL groups, that is, 2,308 shared OTUs, accounting for 84.2 and 96.3% of the total OTUs, respectively (Supplementary Table 2). The rarefaction curve of both PA and FL approached an asymptote (Supplementary Figure 3), indicating sequencing depth was sufficient to capture the bulk of community diversity. Bacterial α-diversity, including Shannon and Chao 1 indices, of the PA lifestyle, was significantly higher than that of the FL lifestyle (Shannon *p* = 0.007, Chao1 *p* < 0.001), In addition, the α-diversity of both lifestyles was significantly higher in autumn than in other seasons (*p* ≤ 0.001) (Figure 1).

**FIGURE 2:**
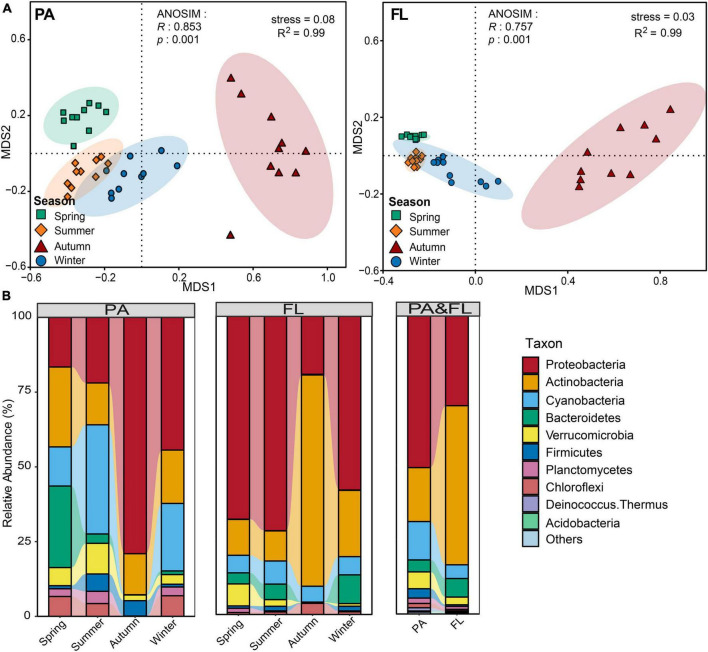
**(A)** Non-metric multidimensional scaling (NMDS) plots showing variations of the particle-attached (PA) and free-living (FL) bacterial communities across different seasons. **(B)** Bacterial taxonomy at the phylum level. Only predominant bacterial phyla are presented; the remaining phyla are assigned to “others.”

Analysis of similarity analysis demonstrated distinct differences in BCCs between PA and FL lifestyles (*R* = 0.23, *p* < 0.001). NMDS and ANOSIM analyses showed that significant temporal variations existed in both PA and FL lifestyles and that the variation of the PA bacterial community structure across seasons was more distinct than that of the FL community (Figure 2A). Partial Mantel tests showed that the variation in bacterial community structure of both PA and FL lifestyles was not significantly correlated with the geographical distance between sampling sites (Figure 5A and Supplementary Figure 4).

**FIGURE 3:**
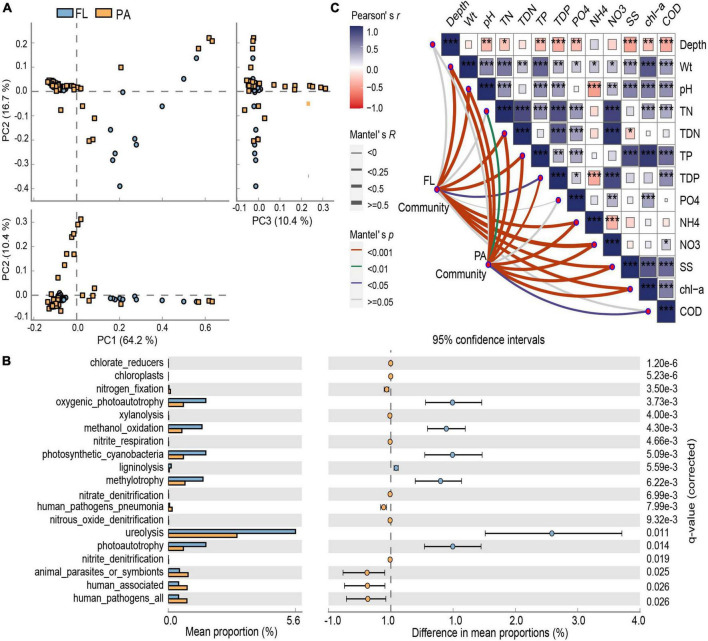
**(A)** Principal component analysis (PCA) plots illustrating comparisons of the bacterial functional profiles. **(B)** Significant differences in functional types between particle-attached (PA) and free-living (FL) lifestyles. **(C)** Pearson’s correlation coefficients of the 13 environmental parameters affecting BCC using Mantel tests. *p < 0.05, **p < 0.01, ***p < 0.001.

**FIGURE 4:**
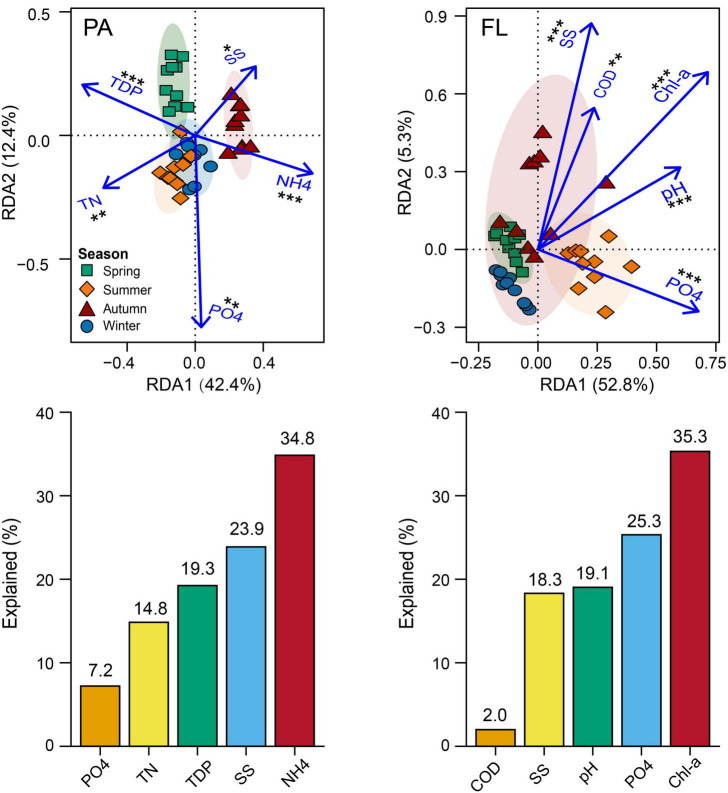
Redundancy analysis (RDA) charts illustrate the relationship between the main environmental parameters and bacterial community compositions (BCCs) of both particle-attached (PA) and free-living (FL) lifestyles. Barcharts are shown below each RDA panel, indicating the contribution of each parameter to the variation of BCC. *p < 0.05, **p < 0.01, ***p < 0.001.

**FIGURE 5:**
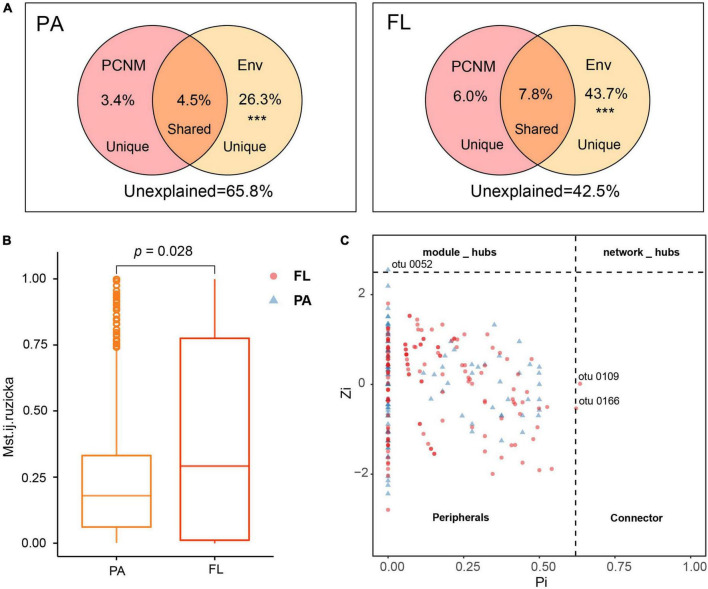
**(A)** Venn chart illustrating the partitioning of variation in bacterial community compositions (BCCs) by environmental parameters (Env.) and by principal coordinates of neighbor matrices (PCNM). ***Permutation test *p*-value = 0.001. **(B)** Modified stochasticity ratio (MST) estimated stochasticity in particle-attached (PA) and free-living (FL) bacterial community assembly. **(C)** *Z*_*i*_-*P*_*i*_ chart illustrating the assignment of nodes according to their topological roles. Each point denotes an OTU in the PA and FL lifestyles.

Taxonomy distribution at the phylum and genus levels and across seasons for both PA and FL lifestyles are shown in Figure 2B and Supplementary Figure 5. Taxa from a total of 17 phyla were observed. Within the PA lifestyle, dominant phyla included *Proteobacteria* (40.5%), *Actinobacteria* (18.1%), and *Cyanobacteria* (18.1%). By contrast, within the FL fraction, the dominant phyla were *Actinobacteria* (54.5%), *Proteobacteria* (28.8%), and *Bacteroidetes* (6.3%). Notably, both the BCC_PA_ and BCC_FL_ in autumn were distinctly different from those in other seasons. In autumn, the dominant phyla of the PA community were *Proteobacteria* (79.0%), *Actinobacteria* (13.7%), and *Firmicutes* (5.1%) while those of FL were *Actinobacteria* (70.9%), *Proteobacteria* (19.6%), and *Bacteroidetes* (5.3%).

Functional annotation of prokaryotic taxa (FAPROTAX) analysis indicated that 13 bacterial metabolic functions were significantly enriched in the PA or FL lifestyles in Lake Tianmu (Figures 3A, B). Across these two communities, 19 functional types had representation higher than would be expected at random. Among them, chlorate-reduction, chloroplasts, nitrogen respiration, nitrogen fixation, nitrate denitrification, and human-associated were significantly higher in the PA lifestyle, while photosynthesis, methylotrophy, methanol oxidation, ligninolysis, and ureolysis were significantly higher in the FL lifestyle (Figure 3B).

### Environmental and spatial factors affecting PA and FL bacterial communities

Mantel test showed a significant correlation between environmental factors and BCC of both lifestyles (PA: *r* = 0.308, *p* < 0.001; FL: *r* = 0.438, *p* < 0.001) (Figure 3C). RDA indicated that 54.8% of the variation in BCC_PA_ was explained by five environmental parameters: NH_4_^+^, SS, TDP, TN, and PO_4_^3–^ (Figure 4A). Among them, NH_4_^+^ was the most important and accounted for 19.1% of the total. Five environmental parameters, Chl-*a*, PO_4_^3–^, pH, SS, and COD, explained 58.1% of the variation in BCC_FL_. Among them, Chl-*a* was the most important and accounted for 20.5% of the total (Figure 4B).

Principal coordinates of neighbor matrices analysis demonstrated that 34.2 and 57.5% of the variation in BCCPA and BCCFL, respectively, could be explained by a combination of environmental parameters and spatial factors (Figure 5A). The proportion of variation explained by pure environmental factors in BCCFL (43.7%) was higher than that in BCCPA (26.3%).

### The assembly processes for PA and FL bacterial communities

Modified stochasticity ratio analysis indicated that t deterministic processes (MST < 0.5) dominated the assembly of both PA and FL bacterial communities (MST PA: 0.241 ± 0.231; FL: 0.386 ± 0.370) (Figure 5B). However, the estimated value of ecological stochasticity was greater in the FL lifestyle than in the PA lifestyle.

### The co-occurrence networks for PA and FL bacterial communities

To elucidate interactions between the bacterial taxon of PA and FL lifestyles, co-occurrence networks for both lifestyles were built. The resulting PA network had 507 edges with 110 nodes remaining, while the FL had 1,420 edges with 146 nodes remaining (Supplementary Figure 7 and Table 1). The topological properties, for example, APL and AvgCC, of the empirical networks of both lifestyles had significantly higher values than those of random networks. Both PA and FL networks were characterized by non-random and high interconnectivity and efficiency as indicated by small values in the “small-world” coefficients (σ > 1) (PA: 2.25; FL: 3.59). The FL network produced higher values of AD, GD, and connectivity compared to the PA network (Table 1), indicating more connections and interactions between the species of the FL lifestyle compared to those of the PA lifestyle. By contrast, analysis of robustness indicated greater stability in the PA network compared to the FL (PA: 0.25 ± 0.05, FL: 0.15 ± 0.06). PA and FL co-occurrence patterns were mostly positively structured (PA: 99.4%: FL: 93.2%).

**TABLE 1:** Major topological attributes of co-occurrence network within particle-attached (PA) and free-living (FL) lifestyles in Lake Tianmu.

	Empirical network										Random network	
	Node	Edge		Modularity	GD	AD	ND	APL	avgCC	σ	APL	avgCC
		Positive	Negative									
PA	110	494 (97.4%)	13 (2.6%)	0.615	0.085	9.218	9	3.184	0.638	2.25	2.323	0.206
FL	146	1,324 (93.2%)	96 (6.8%)	0.586	0.134	19.452	7	2.927	0.679	3.59	2.105	0.136

Node: the number of nodes; Edge: the number of edges; GD, graph density; AD, average degree; ND, network diameter; APL, average path length; avgCC, average clustering coefficient; σ: small-word coefficient, σ = (avgCC/avgCCr)/(APL/APLr), σ > 1 manifests “small-world.”

To compare the ecological functions of keystone taxa in the interaction between PA and FL bacteria, the keystone taxa identified from both groups are shown in Figure 5C. The majority of OTUs categorized in both lifestyles were peripherals (PA, 99.1%; FL, 98.6%) with the majority of correlations falling within their own modules (Figure 5C). However, one module hub (OTU0052, assigned to the genus *Cyanobium* and the phylum *Cyanobacteria*) was identified solely in PA. Two connectors were identified in FL, OTU0109 and OTU0166, identified as *Brevundimonas* and *Sphingomonas*, respectively, both of which belong to the phylum *Proteobacteria* (Figure 5C).

**FIGURE 6:**
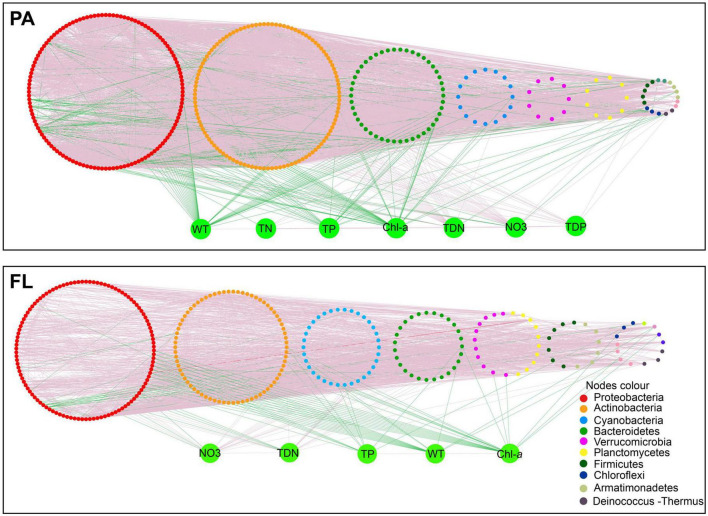
Species–species and species–environment association networks in the particle-attached (PA) and free-living (FL) bacterial lifestyles. A connection denotes a significant correlation coefficients of abs (r) > 0.80 (*p* < 0.01). Different colors denote different phyla. Green lines indicate negative correlations. Red lines indicate positive correlations. Environmental variables: water temperature (WT), total nitrogen (TN), total phosphorus (TP), total dissolved phosphorous (TDP), total dissolved nitrogen (TDN), nitrate (NO_3_^–^), and chlorophyll-*a* (Chl-*a*).

Environmental parameters were imported into the networks to elucidate the relationship between bacteria within each lifestyle and environmental parameters (Figure 6). Numerous significant correlations between bacterial OTUs and environmental parameters (*p* < 0.01) were observed in these two networks. The FL network contained more negative correlations and correlations to more environmental parameters than the PA network (Figure 6 and Supplementary Table 3). Among environmental factors, WT, TP, and Chl-*a* were mostly negatively correlated with the PA and FL networks. Only NO_3_^–^ and TDN exhibited a significant positive correlation with both networks.

## Discussion

### Spatiotemporal variations of PA and FL bacterial communities

Our results demonstrated that α-diversity and BCC_S_ of both PA and FL lifestyles varied by season (Figures 1, 2). This is consistent with the observations of other mesotrophic and eutrophic lakes, including mesotrophic Lake Tiefwaren, Germany (Roesel et al., 2012), and eutrophic Lake Taihu, China (Shen et al., 2022). This temporal variation may be attributed to environmental parameters (e.g., PO_4_^3–^, NH_4_^+^, and Chl-*a*) driven by seasonal alternation (Ortega-Retuerta et al., 2013; He et al., 2018; Yang et al., 2022). In our study, NH_4_^+^ and Chl-*a* were primary contributors to shaping the temporal variation in BCCs of PA and FL communities, respectively (Figure 4). NH_4_^+^ is generally of great importance in determining the distribution of bacterial communities. Large amounts of nitrogen are required for bacteria to synthesize their primary cellular components, including amino sugars, purines, pyrimidines, and amino acids (Diaz-Torres et al., 2022). In addition, Chl-*a* is widely used as an indicator of phytoplankton biomass (Buchan et al., 2014), and the metabolic interaction between bacteria and phytoplankton can lead to either mutual suppression or stimulation of their respective growth. For example, it is well known that phytoplankton competes with bacteria for necessary inorganic nutrients but also supplies organic matter through photosynthesis that is used by bacteria (He et al., 2018). Consistent with this idea, Ortega-Retuerta et al. (2013) reported that Chl-*a* was the primary factor affecting the structure of FL bacterial communities in surface waters from the Mackenzie River to the Beaufort Sea. However, other mechanisms likely influence the seasonal variations of both PA and FL bacterial communities in Lake Tianmu. For example, seasonal differences in precipitation could and likely affect bacterial composition *via* the deposition of soil-associated bacteria *via* runoff leading to a detectable seasonal footprint.

The lowest values in diversity indices for both PA and FL were recorded in autumn (Figure 1). In addition, the greatest seasonal differences in BCC for both lifestyles occur between autumn and the other three seasons (Figure 2). Similar results were observed in PA and FL bacterial communities in eutrophic Lake Taihu (Shen et al., 2022). Three phenomena may help explain these results. The first is environmental filtering, which can be of great importance to bacterial community structure by limiting or removing taxa unable to compete under given conditions. In this case, environmental filtering could have occurred *via* nitrate limitation, as the lowest NO_3_^–^ concentrations were recorded in autumn (Supplementary Figure 2). Second, interspecific interactions were weakest in autumn (Supplementary Figure 7), indicating fewer ecological associations among bacterial communities during this period (Jiao et al., 2020). Finally, as a deep-water lake, the water column of Lake Tianmu is fully mixed by convection in autumn. Loss of stratification can lead to a decline in water quality and notably changes the physical and chemical parameters of the water column leading to large differences in diversity and composition between seasons (Zhang et al., 2003; Zhou et al., 2021).

Functional annotation revealed a rich collection of metabolic types occurring in both PA and FL bacteria as well as highlighting significant differences between the groups (Figure 3B). The genetic capacity for cycling of nitrogen (nitrogen respiration, nitrogen fixation, and nitrate denitrification) was more enriched in PA bacteria than in FL counterparts. Enrichment of the genera *Burkholderia* and *Brevundimonas* (both of *Proteobacteria*) in the PA lifestyle was consistent with this observation. *Burkholderia* has been shown to participate in nitrate reduction and nitrite oxidation in other lakes (Llorens-Mares et al., 2020), while *Brevundimonas* can utilize various organic substrates under aerobic conditions and use nitrate and nitrite as electron acceptors (Kragelund et al., 2005). By contrast, photosynthesis and cycling of carbon (methylotrophy and methanol oxidation) were more enriched in the FL lifestyle. The *cyanobacteria Synechococcus* is a major participant in the global carbon cycle and a contributor to primary productivity (Liu et al., 2015). The functional difference between PA and FL bacteria can be partly explained by the interactions between keystone taxa and other bacterial species (Ma et al., 2020). For example, nitrogen-fixing bacteria supply inorganic nitrogen to *cyanobacteria* in exchange for organic exudates (Yang et al., 2017). In another example, the genera *Sphingopyxis* and *Limnobacter* (of *Proteobacteria*) can degrade microcystins produced by *Cyanobacteria* (Yang et al., 2017; Xu et al., 2021). On a separate note, human-associated pathogenic bacteria were enriched in both the PA and FL fractions in Lake Tianmu, which may pose a health hazard to humans reliant on Lake Tianmu for water (Li et al., 2021).

### The assembly processes for PA and FL bacterial communities in reservoirs

Our results suggest that deterministic processes (i.e., environmental filtering and interspecific interactions) were dominant in bacterial community assembly regardless of PA or FL fractions (Figure 5B). The results indicated that environmental filtering and interspecific interactions contributed more to assembly processes than any stochastic processes (e.g., ecological drift). Nutrients are probably the fundamental contributors governing deterministic processes (Zhao et al., 2017a), and in this study, nutrient species such as NH_4_^+^ and PO_4_^3–^ likely contributed the most. The influence of environmental filtration was greater on the FL bacterial community than on the PA bacterial community (Figure 5A and Supplementary Figure 6). Similar results were observed in eutrophic Lake Taihu (Zhao et al., 2017b). This finding can be partially explained using the size-dispersal hypothesis, which asserts that organisms of smaller size are more vulnerable to environmental filtration (Liu et al., 2019). Another probable explanation is that particulate matter provides a habitat that protects PA bacteria from unfavorable environmental conditions, while FL bacteria are more exposed and more directly affected by environmental parameters such as Chl-*a* and PO_4_^3–^ (Liu et al., 2019). In our study, Chl-*a* and PO_4_^3–^ together accounted for 35.2% of the variation in the FL lifestyle (Figure 4B), and interaction between Chl-*a* and FL bacteria provides additional support and explanation for the above viewpoint (Figure 6).

Despite the dominance of deterministic processes, community assembly within the FL lifestyle was partially shaped by stochastic processes (Figure 5B). This finding is consistent with previous observations in Lake Taihu (Shen et al., 2022). Increased productivity generally increases the relative contribution of stochastic assembly processes within a community (Chase, 2010). In this study, the FL lifestyle had a higher mean abundance (mean = 2.3 ± 1.8 × 10^6^ cells/ml) compared to the PA lifestyle (mean = 1.1 ± 1.0 × 10^6^ cells/ml) (data not shown). Furthermore, Zhou et al. (2014) found that competitive interactions intensified disparities in the structure of communities that originate from stochastic processes once the communities are stimulated. In this case, stimulation was defined as inputs that reduce competition and diminish niche selection (Zhou et al., 2014). As discussed earlier, FL bacteria are more vulnerable to stimulation compared to PA bacteria due to the lack of particle shelter.

### The co-occurrence patterns for PA and FL bacterial communities in reservoirs

In this study, the FL network had more nodes and edges and higher values of AD and GD than did the PA network (Supplementary Figure 7 and Table 1), indicating more ecological connections in the FL network. The FL network contained more ecological associations and more keystone taxa than did the PA network, indicating higher overall network and community complexity in the FL lifestyle (Figure 5C) (Liu S. W. et al., 2022). This conclusion is consistent with our finding of a negative correlation between community diversity and complexity. The lower complexity of the PA lifestyle in this study may be related to its higher α-diversity (Goyal, 2022). Cao et al. (2018) found that habitats with more limited nutrient availability maintained more complex co-occurrence patterns within a bacterial community. Conversely, the abundant nutrient availability typically associated with particle surfaces likely produces a greater diversity of microhabitats to which bacteria in the PA lifestyle may adapt (Mohit et al., 2014; Xu et al., 2018). Nutrient availability was relatively limited in the water column compared with organic particles, which likely induced stronger competition for resources within the FL lifestyle than in the PA lifestyle (Zhou et al., 2014). Thus, our observations in Lake Tianmu are consistent with this described phenomenon. However, tests of robustness indicated that greater stability was maintained in the PA lifestyle than in the FL lifestyle (PA: 0.25 ± 0.05, FL: 0.15 ± 0.06). Similarly, lower connectivity and higher modularity in the PA network suggest that this community was inherently more resistant to environmental perturbation than the FL bacterial community (Yang et al., 2017; Liu S. W. et al., 2022). For this study, the above results from the correlation of complexity and stability analyses might be summarized as bacterial communities with high diversity but low complexity and weak interaction, remaining more stable (Goyal, 2022). There are two intuitive explanations for this phenomenon. First, when species in a community interact strongly, negative effects on stability are more likely to occur when a valuable ecological function is lost (Huelsmann and Ackermann, 2022). Second, communities with high diversity likely comprise multiple species with redundant functions but possibly differing abilities to withstand perturbation, thus if the function of one species is disturbed, other species are available to replace that function (Huelsmann and Ackermann, 2022).

Keystone taxa are of great importance in community structure and function due to their distinctive and integral biological interactions (Liu S. W. et al., 2022). Two connectors (OTU0109 and OTU0166 assigned to *Proteobacteria*) were identified in the FL network, while only one module hub (OTU0052; assigned to *Cyanobacteria*) was identified in the PA network (Figure 5C), indicating that keystone taxa in PA and FL networks played distinct topological roles. As the keystone taxon in the PA network, *Cyanobacteria* can provide nutrients and organic substrates used by heterotrophic bacteria through either photosynthesis or by serving as prey to antagonistic bacteria (Yang et al., 2017). In the FL network, OUT0109 and OUT0166 were assigned to the genera *Brevundimonas* and *Sphingomonas*, respectively, but both played the same topological role as a connector. Previous studies have reported that *Brevundimonas* can serve in the functions of biosorption of pollutants, hydrolysis of lactose, degradation of quinoline, and the promotion of growth of *Chlorella* (Yang et al., 2017). *Sphingomonas* are capable of degrading various complex organics (Cheng et al., 2019). While OTU0052 (PA) had the capacity to contribute to the production and cycling of nutrients, OUT0109 and OTU0166 (FL) had the capacities to degrade pollutants and repair the environment, indicating that keystone taxa of PA and FL bacterial communities engaged in distinctly different ecological functions. The high abundance of *Cyanobacteria* and *Proteobacteria* in both PA and FL networks (Figure 2B) further supports their indispensable function in the Lake Tianmu ecosystem. A note of caution is due here since the mentioned functions are inferred from 16S sequencing, where bacteria with similar or even identical 16S sequences may have very different putative functions.

A greater number of negative interactions were observed in the FL network compared to the PA network (Supplementary Table 3). This is consistent with the fact that the structure of FL bacterial communities was significantly correlated to more environmental parameters than were the PA bacterial communities (Figure 6), which suggests that environmental filtration had a greater impact on the FL lifestyle than on the PA lifestyle. TDN and NO_3_^–^ had significant positive correlations with OTUs in both lifestyles, indicating, that high N concentrations favored species in both networks. By contrast, WT, TP, and Chl-*a* were negatively correlated with OTUs in both networks. WT exhibited a strong control over co-occurrence patterns, probably due to its effects on multiple biotic and abiotic factors (Jiao et al., 2020) including the growth and respiration of bacteria (Martinez-Garcia et al., 2022). High concentrations of TP and chlorophyll indicate continued eutrophication of the lake, which is frequently associated with the release of harmful algal toxins and can lead to hypoxia as heterotrophic bacteria consume oxygen while degrading excess algal biomass (Yue et al., 2021). Previous studies reported that a high concentration of Chl-*a* and TP in Lake Tianmu has contributed greatly to the recent decline in water quality (Yang et al., 2021). Our results provide ecological insights into distinct interactions between environmental parameters and PA and FL bacterial communities in Lake Tianmu.

## Conclusion

We found obvious temporal and spatial variations in the PA and FL bacterial communities of Lake Tianmu, an artificial reservoir in east-central China. Putative genetic capacity for cycling of nitrogen and carbon was abundant in PA and FL communities, respectively. Deterministic processes were the primary contributors in shaping the assembly of bacterial communities for both lifestyles. However, the structures of PA and FL bacterial communities were shaped by different environmental parameters. Bacterial diversity and complex interspecies interactions drove the difference in co-occurrence between PA and FL lifestyles. The PA network contained one module hub assigned to *Cyanobacteria* with the network function of mediating biogeochemical cycles. The FL network contained two connectors assigned to *Brevundimonas and Sphingomonas* with the network function of degradation of pollutants. PA bacteria were more resistant in the face of changing environmental conditions. Our findings highlight the similarities and differences in assembly processes and responses to environmental variation for PA and FL bacterial communities in a drinking water reservoir.

## Data availability statement

The datasets presented in this study can be found in online repositories. The names of the repository/repositories and accession number(s) can be found in the article/Supplementary material.

## Author contributions

BY: methodology, writing—original draft, and visualization. ZS: project administration. GX: investigation. KS: writing—review and editing. XT: funding acquisition. All authors contributed to the article and approved the submitted version.

## References

[B1] Borcard D. Legendre P. (2002). All-scale spatial analysis of ecological data by means of principal coordinates of neighbour matrices. *Ecol. Model.* 153 51–68.

[B2] Buchan A. LeCleir G. R. Gulvik C. A. Gonzalez J. M. (2014). Master recyclers: Features and functions of bacteria associated with phytoplankton blooms. *Nat. Rev. Microbiol.* 12 686–698. 10.1038/nrmicro3326 25134618

[B3] Cao X. Y. Zhao D. Y. Xu H. M. Rui H. Zeng J. Yu Z. B. (2018). Heterogeneity of interactions of microbial communities in regions of Taihu Lake with different nutrient loadings: A network analysis. *Sci. Rep.* 8:8890. 10.1038/s41598-018-27172-z 29891905 PMC5995825

[B4] Chaffron S. Rehrauer H. Pernthaler J. von Mering C. (2010). A global network of coexisting microbes from environmental and whole-genome sequence data. *Genome Res.* 20 947–959. 10.1101/gr.104521.109 20458099 PMC2892096

[B5] Chase J. M. (2010). Stochastic community assembly causes higher biodiversity in more productive environments. *Science* 328 1388–1391. 10.1126/science.1187820 20508088

[B6] Cheng M. G. Yan X. He J. Qiu J. G. Chen Q. (2019). Comparative genome analysis reveals the evolution of chloroacetanilide herbicide mineralization in Sphingomonas wittichii DC-6. *Arch. Microbiol.* 201 907–918. 10.1007/s00203-019-01660-w 30997539

[B7] Christensen H. (2018). *Introduction to bioinformatics in microbiology.* Cham: Springer.

[B8] Crespo B. G. Pommier T. Fernandez-Gomez B. Pedros-Alio C. (2013). Taxonomic composition of the particle-attached and free-living bacterial assemblages in the Northwest Mediterranean Sea analyzed by pyrosequencing of the 16S rRNA. *Microbiologyopen* 2 541–552. 10.1002/mbo3.92 23723056 PMC3948605

[B9] Deng Y. Jiang Y. H. Yang Y. F. He Z. L. Luo F. Zhou J. Z. (2012). Molecular ecological network analyses. *BMC Bioinform.* 13:113. 10.1186/1471-2105-13-113 22646978 PMC3428680

[B10] Diaz-Torres O. Lugo-Melchor O. Y. de Anda J. Pacheco A. Yebra-Montes C. Gradilla-Hernandez M. S. (2022). Bacterial dynamics and their influence on the biogeochemical cycles in a subtropical hypereutrophic lake during the rainy season. *Front. Microbiol.* 13:832477. 10.3389/fmicb.2022.832477 35479621 PMC9037096

[B11] Fadrosh D. W. Ma B. Gajer P. Sengamalay N. Ott S. Brotman R. M. (2014). An improved dual-indexing approach for multiplexed 16S rRNA gene sequencing on the Illumina MiSeq platform. *Microbiome* 2:6. 10.1186/2049-2618-2-6 24558975 PMC3940169

[B12] Goyal A. (2022). How diverse ecosystems remain stable. *Nat. Ecol. Evol.* 6 667–668. 10.1038/s41559-022-01758-3 35484220

[B13] Grossart H. P. (2010). Ecological consequences of bacterioplankton lifestyles: Changes in concepts are needed. *Environ. Microbiol. Rep.* 2 706–714. 10.1111/j.1758-2229.2010.00179.x 23766274

[B14] He J. Y. Wang K. Xiong J. B. Guo A. N. Zhang D. M. Fei Y. J. (2018). Drivers of coastal bacterioplankton community diversity and structure along a nutrient gradient in the East China Sea. *J. Oceanol. Limnol.* 36 329–340. 10.1007/s00343-017-6104-7

[B15] Huelsmann M. Ackermann M. (2022). Community instability in the microbial world. *Science* 378 29–30. 10.1126/science.ade2516 36201571

[B16] Jiao C. C. Zhao D. Y. Zeng J. Guo L. Yu Z. B. (2020). Disentangling the seasonal co-occurrence patterns and ecological stochasticity of planktonic and benthic bacterial communities within multiple lakes. *Sci. Total Environ.* 740:140010. 10.1016/j.scitotenv.2020.140010 32563874

[B17] Jin X. C. Tu Q. Y. (1990). *The standard methods for observation and analysis in lake eutrophication*, Vol. 240. Beijing: Chinese Environmental Science Press, 138–272.

[B18] Kragelund C. Nielsen J. L. Thomsen T. R. Nielsen P. H. (2005). Ecophysiology of the filamentous Alphaproteobaeterium Meganema perideroedes in activated sludge. *FEMS Microbiol. Ecol.* 54 111–122. 10.1016/j.femsec.2005.03.002 16329977

[B19] Lai J. S. Zou Y. Zhang J. L. Peres-Neto P. R. (2022). Generalizing hierarchical and variation partitioning in multiple regression and canonical analyses using the rdacca.hp R package. *Methods Ecol. Evol.* 13 782–788.

[B20] Lapoussiere A. Michel C. Starr M. Gosselin M. Poulin M. (2011). Role of free-living and particle-attached bacteria in the recycling and export of organic material in the Hudson Bay system. *J. Mar. Syst.* 88 434–445. 10.1016/j.jmarsys.2010.12.003

[B21] Li C. C. Wang L. F. Ji S. P. Chang M. J. Wang L. F. Gan Y. D. (2021). The ecology of the plastisphere: Microbial composition, function, assembly, and network in the freshwater and seawater ecosystems. *Water Res.* 202:117428. 10.1016/j.watres.2021.117428 34303166

[B22] Liao J. Cao X. Zhao L. Wang J. Gao Z. Wang M. C. (2016). The importance of neutral and niche processes for bacterial community assembly differs between habitat generalists and specialists. *FEMS Microbiol. Ecol.* 92:fiw174. 10.1093/femsec/fiw174 27543321

[B23] Liu C. C. Xiang L. Y. Xu R. Zhang H. F. (2015). Relationship between Synechococcus distribution and environmental factors in the East China Sea in summer. *J. Shanghai Ocean Univ.* 24 886–893.

[B24] Liu J. M. Wang X. L. Liu J. Liu X. Y. Zhang X. H. Liu J. W. (2022). Comparison of assembly process and co-occurrence pattern between planktonic and benthic microbial communities in the Bohai Sea. *Front. Microbiol.* 13:1003623. 10.3389/fmicb.2022.1003623 36386657 PMC9641972

[B25] Liu K. S. Hou J. Z. Liu Y. Q. Hu A. Y. Wang M. D. Wang F. (2019). Biogeography of the free-living and particle-attached bacteria in Tibetan lakes. *FEMS Microbiol. Ecol.* 95:fiz088. 10.1093/femsec/fiz088 31183497

[B26] Liu S. W. Yu H. Yu Y. H. Huang J. Zhou Z. Y. Zeng J. X. (2022). Ecological stability of microbial communities in Lake Donghu regulated by keystone taxa. *Ecol. Indic.* 136:108695. 10.1016/j.ecolind.2022.108695

[B27] Llorens-Mares T. Catalan J. Casamayor E. O. (2020). Taxonomy and functional interactions in upper and bottom waters of an oligotrophic high-mountain deep lake (Redon, Pyrenees) unveiled by microbial metagenomics. *Sci. Total Environ.* 707:135929. 10.1016/j.scitotenv.2019.135929 31863999

[B28] Louca S. Jacques S. M. S. Pires A. P. F. Leal J. S. Srivastava D. S. Parfrey L. W. (2018). High taxonomic variability despite stable functional structure across microbial communities. *Nat. Ecol. Evol.* 1:15. 10.1038/s41559-016-0015 28812567

[B29] Louca S. Parfrey L. W. Doebeli M. (2016). Decoupling function and taxonomy in the global ocean microbiome. *Science* 353 1272–1277. 10.1126/science.aaf4507 27634532

[B30] Ma Q. Liu S. W. Li S. Z. Hu J. B. Tang M. Y. Sun Y. Q. (2020). Removal of malodorant skatole by two enriched microbial consortia: Performance, dynamic, function prediction and bacteria isolation. *Sci. Total Environ.* 725:138416. 10.1016/j.scitotenv.2020.138416 32302841

[B31] Martinez-Garcia S. Bunse C. Pontiller B. Baltar F. Israelsson S. Fridolfsson E. (2022). Seasonal dynamics in carbon cycling of marine bacterioplankton are lifestyle dependent. *Front. Microbiol.* 13:834675. 10.3389/fmicb.2022.834675 36212867 PMC9533715

[B32] Mohit V. Archambault P. Toupoint N. Lovejoy C. (2014). Phylogenetic differences in attached and free-living bacterial communities in a temperate coastal lagoon during summer, revealed via high-throughput 16S rRNA gene sequencing. *Appl. Environ. Microbiol.* 80 2071–2083. 10.1128/AEM.02916-13 24463966 PMC3993158

[B33] Nemergut D. R. Schmidt S. K. Fukami T. O’Neill S. P. Bilinski T. M. Stanish L. F. (2013). Patterns and processes of microbial community assembly. *Microbiol. Mol. Biol. Rev.* 77 342–356. 10.1128/mmbr.00051-12 24006468 PMC3811611

[B34] Ning D. L. Deng Y. Tiedje J. M. Zhou J. Z. (2019). A general framework for quantitatively assessing ecological stochasticity. *Proc. Natl. Acad. Sci. U.S.A.* 116 16892–16898. 10.1073/pnas.1904623116 31391302 PMC6708315

[B35] Ortega-Retuerta E. Joux F. Jeffrey W. H. Ghiglione J. F. (2013). Spatial variability of particle-attached and free-living bacterial diversity in surface waters from the Mackenzie River to the Beaufort Sea (Canadian Arctic). *Biogeosciences* 10 2747–2759. 10.5194/bg-10-2747-2013

[B36] Parks D. H. Tyson G. W. Hugenholtz P. Beiko R. G. (2014). STAMP: Statistical analysis of taxonomic and functional profiles. *Bioinformatics* 30 3123–3124. 10.1093/bioinformatics/btu494 25061070 PMC4609014

[B37] Parveen B. Reveilliez J. P. Mary I. Ravet V. Bronner G. Mangot J. F. (2011). Diversity and dynamics of free-living and particle-associated Betaproteobacteria and Actinobacteria in relation to phytoplankton and zooplankton communities. *FEMS Microbiol. Ecol.* 77 461–476. 10.1111/j.1574-6941.2011.01130.x 21585402

[B38] Quast C. Pruesse E. Yilmaz P. Gerken J. Glckner F. O. (2012). The SILVA ribosomal RNA gene database project: Improved data processing and web-based tools. *Nucleic Acids Res.* 41 D590–D596. 10.1093/nar/gks1219 23193283 PMC3531112

[B39] Roesel S. Allgaier M. Grossart H. P. (2012). Long-term characterization of free-living and particle-associated bacterial communities in lake tiefwaren reveals distinct seasonal patterns. *Microb. Ecol.* 64 571–583. 10.1007/s00248-012-0049-3 22526401

[B40] Roeske K. Sachse R. Scheerer C. Roeske I. (2012). Microbial diversity and composition of the sediment in the drinking water reservoir Saidenbach (Saxonia, Germany). *Syst. Appl. Microbiol.* 35 35–44. 10.1016/j.syapm.2011.09.002 22154008

[B41] Shen Z. Xie G. J. Zhang Y. Q. Yu B. B. Shao K. Q. Gao G. (2022). Similar assembly mechanisms but distinct co-occurrence patterns of free-living vs. particle-attached bacterial communities across different habitats and seasons in shallow, eutrophic Lake Taihu. *Environ. Pollut.* 314:120305. 10.1016/j.envpol.2022.120305 36181942

[B42] Stegen J. C. Lin X. J. Fredrickson J. K. Konopka A. E. (2015). Estimating and mapping ecological processes influencing microbial community assembly. *Front. Microbiol.* 6:370. 10.3389/fmicb.2015.00370 25983725 PMC4416444

[B43] Tang X. M. Li L. L. Shao K. Q. Wang B. W. Cai X. L. Zhang L. (2015). Pyrosequencing analysis of free-living and attached bacterial communities in Meiliang Bay, Lake Taihu, a large eutrophic shallow lake in China. *Can. J. Microbiol.* 61 22–31. 10.1139/cjm-2014-0503 25496473

[B44] Tang X. Xie G. Shao K. Hu Y. Cai J. Bai C. (2020). Contrast diversity patterns and processes of microbial community assembly in a river-lake continuum across a catchment scale in northwestern China. *Environ. Microbiome* 15:10. 10.1186/s40793-020-00356-9 33902721 PMC8066441

[B45] Tang X. Xie G. Shao K. Tian W. Gao G. Qin B. (2021). Aquatic bacterial diversity, community composition and assembly in the semi-arid inner mongolia plateau: Combined effects of salinity and nutrient levels. *Microorganisms* 9:208. 10.3390/microorganisms9020208 33498349 PMC7909399

[B46] Xie G. J. Tang X. M. Shao K. Q. Zhu G. W. Gao G. (2021). Bacterial diversity, community composition and metabolic function in Lake Tianmuhu and its dammed river: Effects of domestic wastewater and damming. *Ecotoxicol. Environ. Saf.* 213:112069. 10.1016/j.ecoenv.2021.112069 33631636

[B47] Xu H. M. Zhao D. Y. Huang R. Cao X. Y. Zeng J. Yu Z. B. (2018). Contrasting network features between free-living and particle-attached bacterial communities in Taihu Lake. *Microb. Ecol.* 76 303–313. 10.1007/s00248-017-1131-7 29318328

[B48] Xu S. He C. Song S. Q. Li C. W. (2021). Spatiotemporal dynamics of marine microbial communities following a Phaeocystis bloom: Biogeography and co-occurrence patterns. *Environ. Microbiol. Rep.* 13 294–308. 10.1111/1758-2229.12929 33527743

[B49] Yang C. Y. Wang Q. Simon P. N. Liu J. Y. Liu L. C. Dai X. Z. (2017). Distinct network interactions in particle-associated and free-living bacterial communities during a *Microcystis aeruginosa* bloom in a Plateau Lake. *Front. Microbiol.* 8:1202. 10.3389/fmicb.2017.01202 28713340 PMC5492469

[B50] Yang W. B. Duan W. X. Cui Y. Zhu G. W. Wu T. H. Xu H. (2021). Long-term changes and drivers of ecological security in Shahe Reservoir, China. *Huan Jing Ke Xue* 42 4739–4752. 10.13227/j.hjkx.202101125 34581116

[B51] Yang Y. Chen C. Wang J. Y. Xu T. (2022). Characterizing free-living and particle-attached bacterial communities of a canyon river reservoir on the Yungui Plateau, China. *Front. Microbiol.* 13:986637. 10.3389/fmicb.2022.986637 36118241 PMC9470832

[B52] Yuan H. T. Li T. C. Li H. F. Wang C. Li L. Lin X. (2021). Diversity distribution, driving factors and assembly mechanisms of free-living and particle-associated bacterial communities at a subtropical marginal sea. *Microorganisms* 9:2445. 10.3390/microorganisms9122445 34946047 PMC8704526

[B53] Yue Y. Cai L. Tang Y. Zhang Y. Yang M. Wang F. (2021). Vertical distribution of bacterial community in water columns of reservoirs with different trophic conditions during thermal stratification. *Front. Environ. Sci.* 9:632089. 10.3389/fenvs.2021.632089

[B54] Zhang H. H. Huang T. L. Chen S. N. Guo L. Yang X. Liu T. T. (2013). Spatial pattern of bacterial community functional diversity in a drinking water reservoir, Shaanxi Province, Northwest China. *J. Pure Appl. Microbiol.* 7 1647–1654.

[B55] Zhang H. H. Huang T. L. Chen S. N. Yang X. Lv K. Sekar R. (2015). Abundance and diversity of bacteria in oxygen minimum drinking water reservoir sediments studied by quantitative PCR and pyrosequencing. *Microb. Ecol.* 69 618–629. 10.1007/s00248-014-0539-6 25502074

[B56] Zhang T. Xu S. Yan R. Wang R. Gao Y. Kong M. (2022). Similar geographic patterns but distinct assembly processes of abundant and rare bacterioplankton communities in river networks of the Taihu Basin. *Water Res.* 211:118057. 10.1016/j.watres.2022.118057 35066261

[B57] Zhang Y. L. Chen W. M. Yang D. T. Jiang J. (2003). The current water environment of Tianmuhu Lake and countermeasures for the sustainable development of the ecological tourism. *Ecol. Environ.* 12 405–408.

[B58] Zhao D. Y. Cao X. Y. Huang R. Zeng J. Shen F. Xu H. M. (2017a). The heterogeneity of composition and assembly processes of the microbial community between different nutrient loading lake zones in Taihu Lake. *Appl. Microbiol. Biotechnol.* 101 5913–5923. 10.1007/s00253-017-8327-0 28523397

[B59] Zhao D. Y. Xu H. M. Zeng J. Cao X. Y. Huang R. Shen F. (2017b). Community composition and assembly processes of the free-living and particle-attached bacteria in Taihu Lake. *FEMS Microbiol. Ecol.* 93:fix062. 10.1093/femsec/fix062 28498948

[B60] Zhou J. Deng Y. Zhang P. Xue K. Liang Y. Van Nostrand J. D. (2014). Stochasticity, succession, and environmental perturbations in a fluidic ecosystem. *Proc. Natl. Acad. Sci. U.S.A.* 111 E836–E845. 10.1073/pnas.1324044111 24550501 PMC3948316

[B61] Zhou L. Zhou Y. Q. Zhang Y. L. Zhu G. W. (2021). Characterizing sources and composition of chromophoric dissolved organic matter in a key drinking water reservoir lake Tianmu. *Huan Jing Ke Xue* 42 3709–3718. 10.13227/j.hjkx.202012280 34309257

