# Supplementary material for: Spatiotemporal variations, assembly processes, and co-occurrence patterns of particle-attached and free-living bacteria in a large drinking water reservoir in China
Source: Front Microbiol. 2023 Jan 19;13:1056147. doi: 10.3389/fmicb.2022.1056147 (PMC9892854; doi:10.3389/fmicb.2022.1056147)
Supplement: Supplementary file 1 [file Data_Sheet_1.docx]

Supplementary Materials

# Supplementary Tables

**Table S1.** Definitions and descriptions of terms appearing in the article.

| Terms | Definitions and Interpretation |
| --- | --- |
| Assembly processes | Two ecological process theories (based on niche process theory and neutral process theory) jointly describe the assembly mechanism of bacterial communities. Niche theory assumes that certain factors such as species characteristics, interactions between species and environmental conditions control community structure and metabolic function. That is, microbial communities are formed by certain biological factors (species interaction, such as competition and predation) and abiotic factors (environmental factors, such as pH and temperature), which are caused by different habitat preferences and adaptations of microorganisms. The neutral process theory assumes that the loss and increase of microorganisms in groups show a random balance, that is, random processes (birth, death, migration, species formation, diffusion limitation) shape the microbial community structure. |
| Environmental filtration | Selection of bacterial communities by abiotic environmental factors (such as pH and temperature). |
| Deterministic processes | Deterministic processes are caused by the predictable filtration of species by ecological selection imposed by biological and abiotic factors. They affect the adaptation of organisms, thus determining the composition and relative abundance of species. The deterministic process includes the selection of abiotic environmental factors and the antagonism and synergy between species. |
| Stochastic processes | Stochastic process involves random birth, death, probability diffusion and random change of relative abundance of species, not the result of fitness determined by environment. |
| Graph density (GD) | Graph density (GD) refers to the frequency ratio of the actual edge to the possible edge, which reflects the cohesiveness of the network. |
| Average degree (AD) | The average degree (AD) is the average of the degrees of all nodes in the network, which can reflect the overall connectivity of the network. |
| Average clustering coefficient (AvgCC) | The triangular part formed by a single node and its surroundings is called clustering coefficient, which is similar to the number of neighbor parts of the node. The average clustering coefficient of the network reflects the universality of clustering connectivity around a single node. |
| Average path length (APL) | Shorter average path lengths (APL) within a network suggest greater efficiency in the transferring of information, energy, and matter between taxa, whereas higher modules indicate intense competition between species, which tends to reduce network stability |

**Table S2.** OTU (97% similarity) distribution patterns in particle-attached (PA) and free-living (FL) bacterial communities.

|  | Numbers and percentages of shared OTUs | | Number and percentage (%) of unique OTUs | | | |
| --- | --- | --- | --- | --- | --- | --- |
|  |  |  | PA | | FL | |
| OTUs distribution | Numbers | Percentages (%) | Numbers | Percentages (%) | Numbers | Percentages (%) |
| Overall bacteria | 2308 | 81.5 | 433 | 15.3 | 90 | 3.2 |
| Proteobacteria | 1070 | 83.4 | 169 | 13.2 | 44 | 3.4 |
| Actinobacteria | 462 | 89.9 | 37 | 7.2 | 15 | 2.9 |
| Bacteroidetes | 312 | 87.9 | 23 | 6.5 | 20 | 5.6 |
| Firmicutes | 130 | 76.5 | 34 | 20.0 | 6 | 3.5 |
| Verrucomicrobia | 75 | 90.4 | 7 | 8.43 | 1 | 1.2 |
| Cyanobacteria | 61 | 73.5 | 22 | 26.5 | 0 | 0 |
| Acidobacteria | 47 | 37.9 | 75 | 60.5 | 2 | 1.6 |
| Chloroflexi | 37 | 44.6 | 46 | 55.4 | 0 | 0 |
| Planctomycetes | 22 | 91.7 | 2 | 8.3 | 0 | 0 |
| Nitrospirae | 21 | 70.0 | 9 | 30.0 | 0 | 0 |
| Deinococcus-Thermus | 17 | 94.4 | 1 | 5.6 | 0 | 0 |
| Others | 182 | 94.8 | 8 | 4.2 | 2 | 1.0 |

**Table S3.** Number of positive/negative links between environmental variables and species in species-environment association networks in PA and FL bacterial communities. Environmental variables: water temperature (WT), total nitrogen (TN), total phosphorus (TP), total dissolved phosphorous (TDP), total dissolved nitrogen (TDN), nitrate (NO_3_^-^) and chlorophyll-*a* (Chl-*a*).

|  | PA | |  | FL | |
| --- | --- | --- | --- | --- | --- |
|  | Positive links | Negative links |  | Positive links | Negative links |
| Total | 44 | 60 |  | 66 | 106 |
| WT | 0 | 17 |  | 1 | 37 |
| TN | 4 | 0 |  | 4 | 1 |
| TDN | 18 | 4 |  | 26 | 3 |
| TP | 0 | 11 |  | 0 | 21 |
| TDP | 4 | 0 |  | 12 | 0 |
| NO3 | 18 | 3 |  | 23 | 4 |
| Chl-*a* | 0 | 25 |  | 0 | 40 |

# Supplementary Figures

**
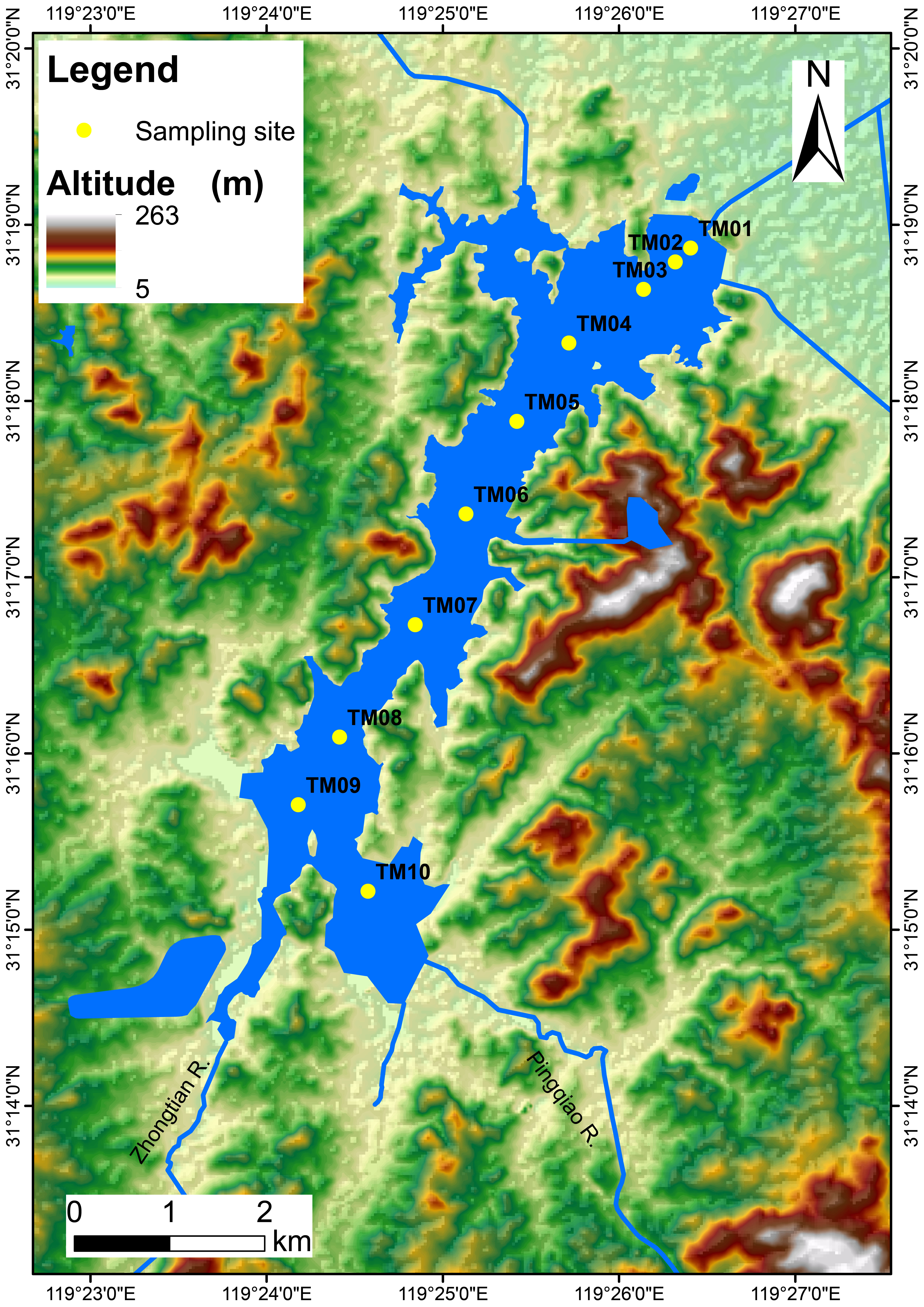
**

**Figure S1.** Location of sampling sites in Lake Tianmu.


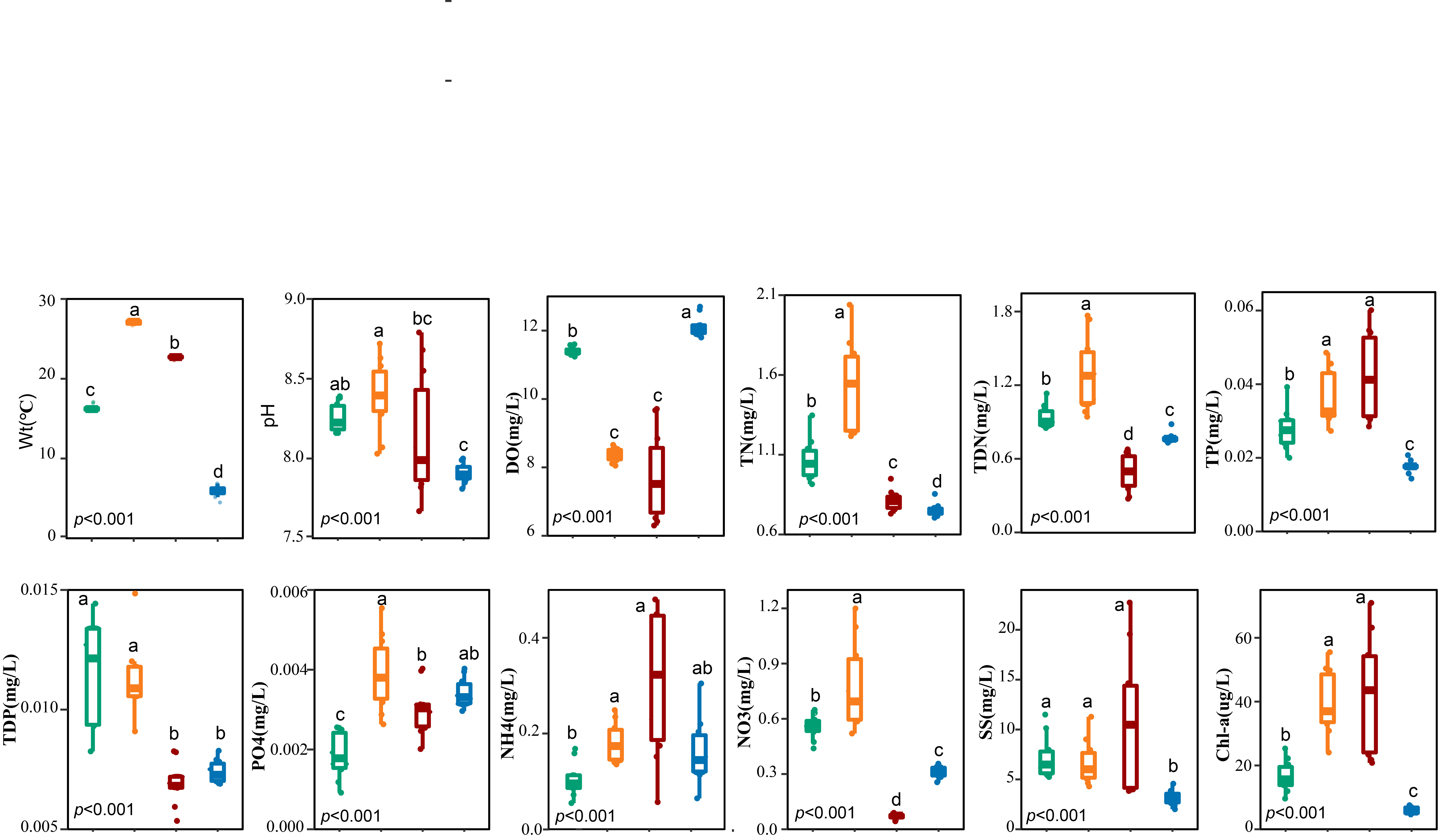


**Figure S2.** Comparison of environmental parameters in Lake Tianmu. Kruskal-Wallis test was performed to examine differences among seasons with *p*-value presented at the bottom of each panel.


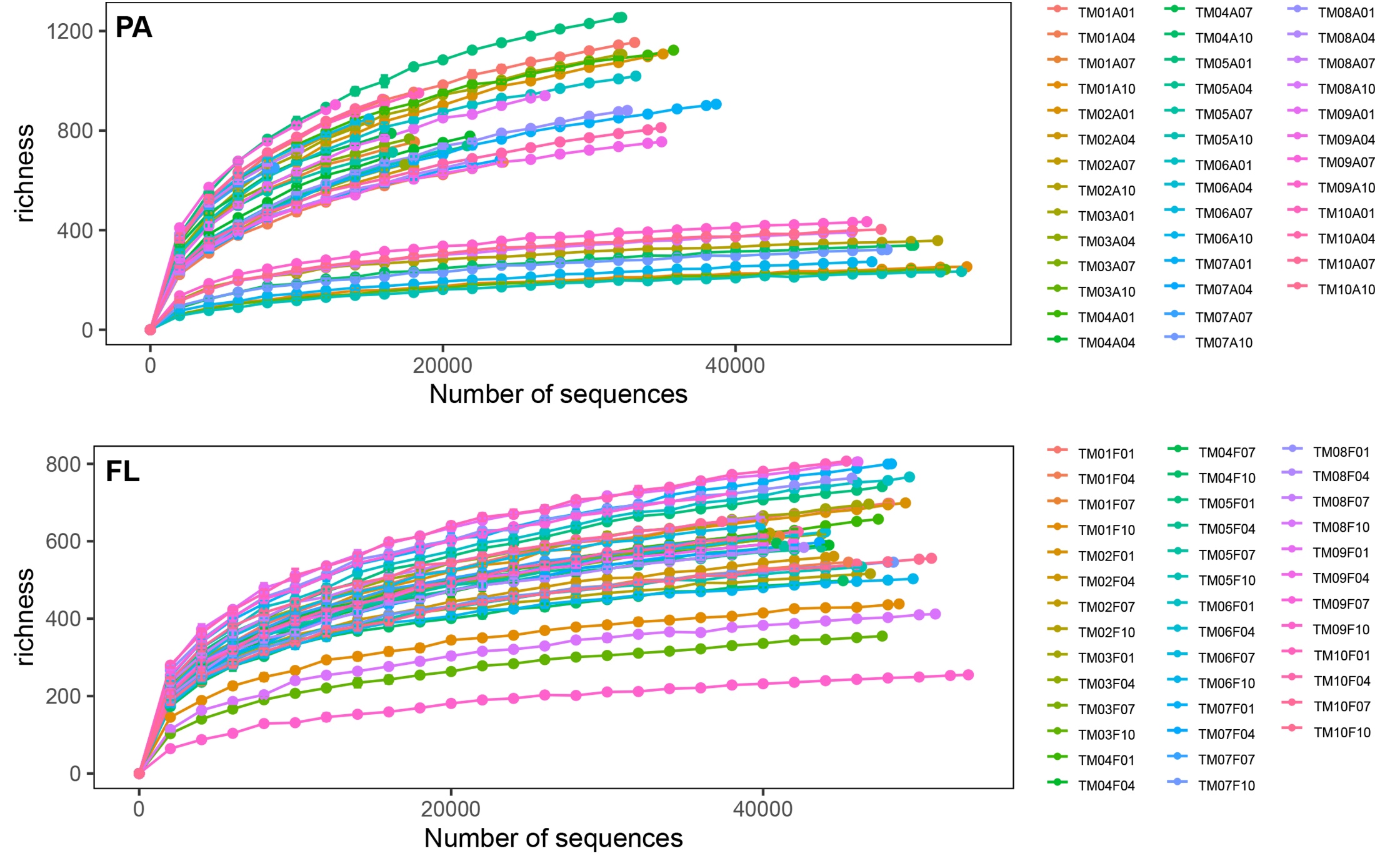


**Figure S3.** Rarefaction curves of species richness for 80 samples (PA and FL).


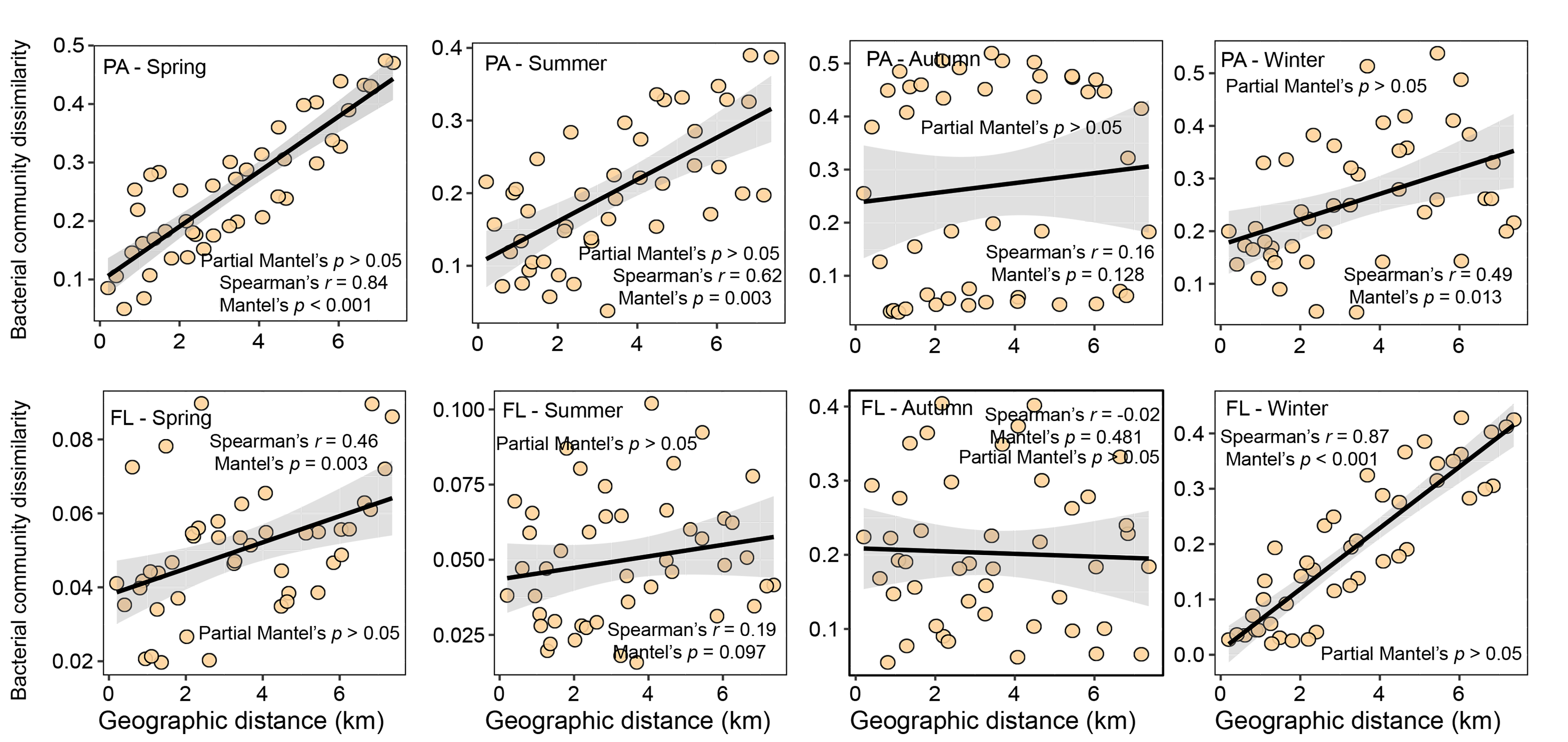


**Figure S4.** Spearman’s rank correlations between bacterial community dissimilarity (Bray-Curtis distance) and geographical distance in PA and FL lifestyles and four seasons. Note: *r* and *p* refer to the rank correlations and statistical significance, respectively.


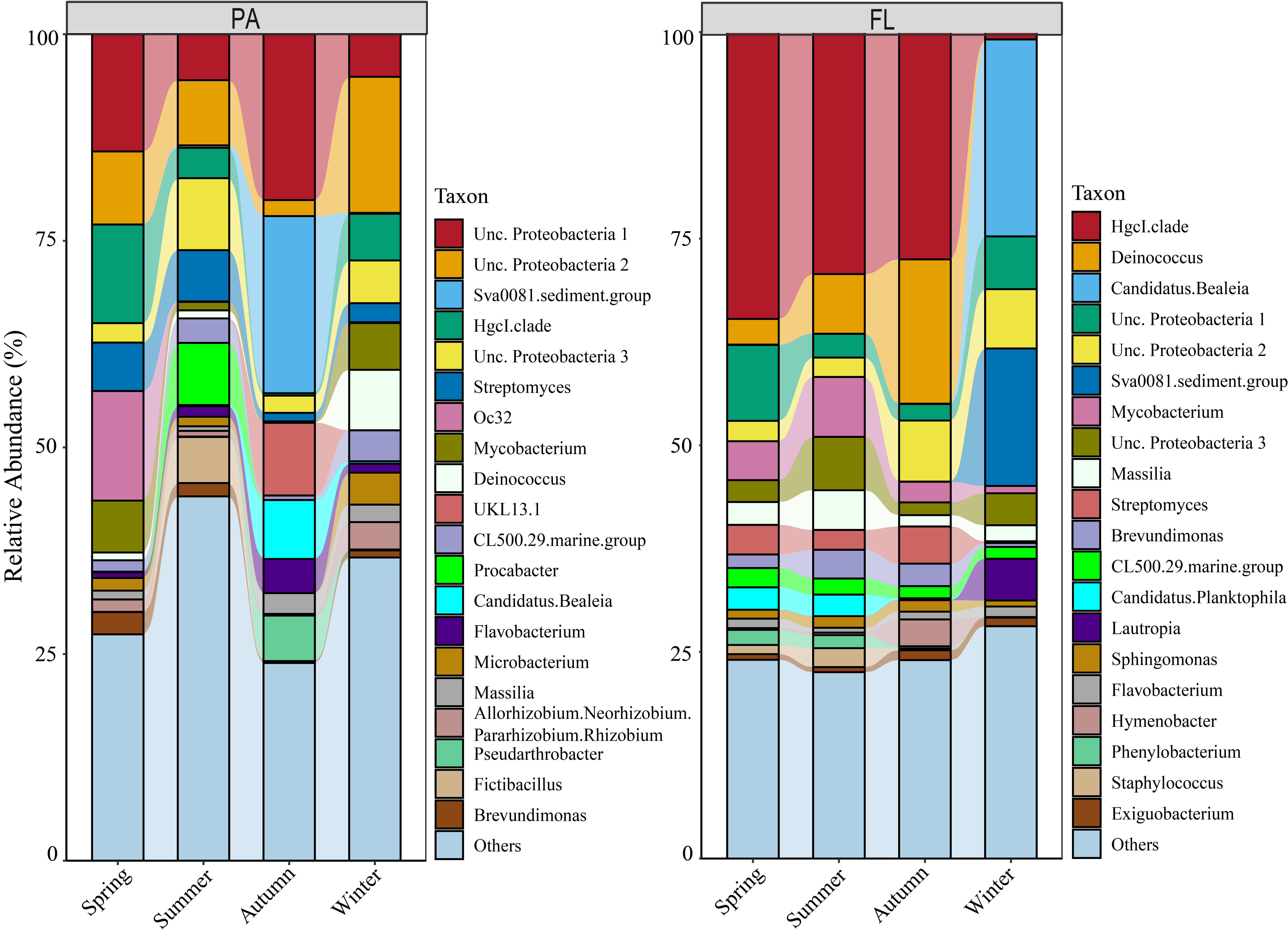


**Figure S5.** Bacterial taxonomy at the genus level. Only predominant bacterial genera are presented; remaining genera are assigned to ‘others’.


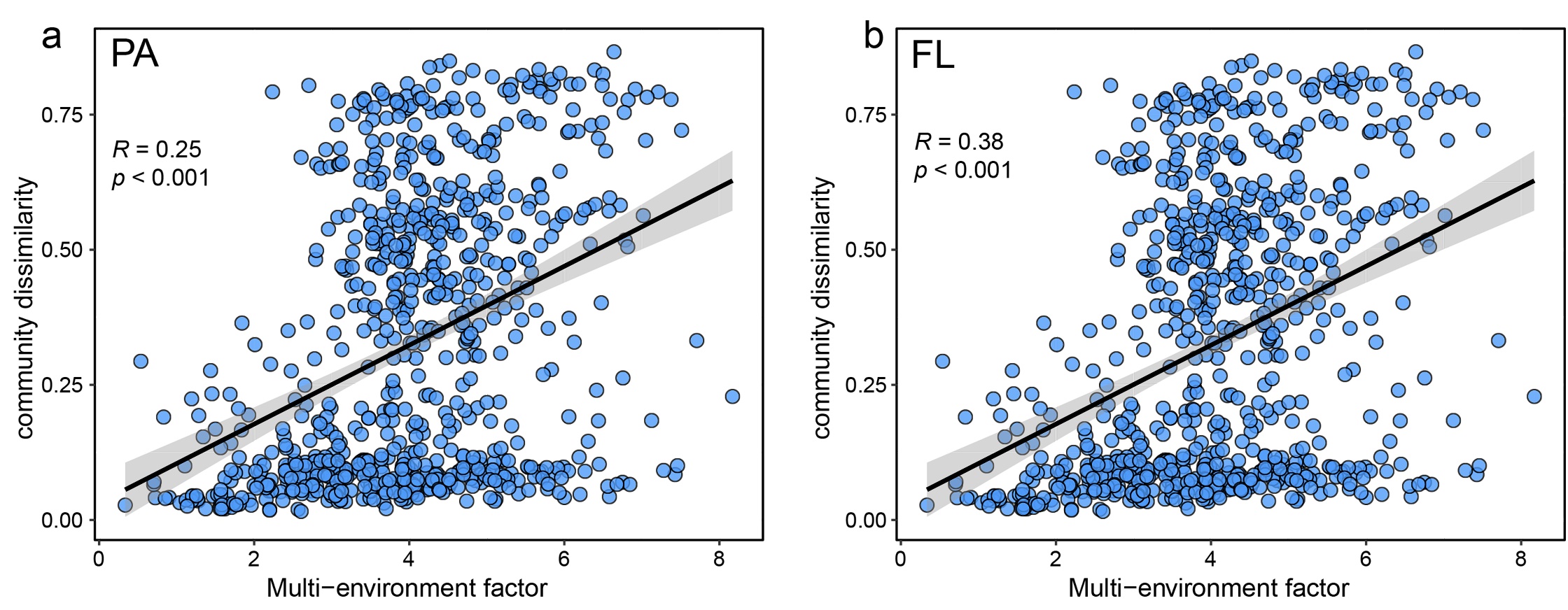


**Figure S6.** Spearman’s rank correlations between bacterial community dissimilarity (Bray-Curtis distance) and environmental dissimilarity (Euclidean distance) in PA and FL lifestyles. Note: *R* and *p* refer to the rank correlations and statistical significance, respectively.


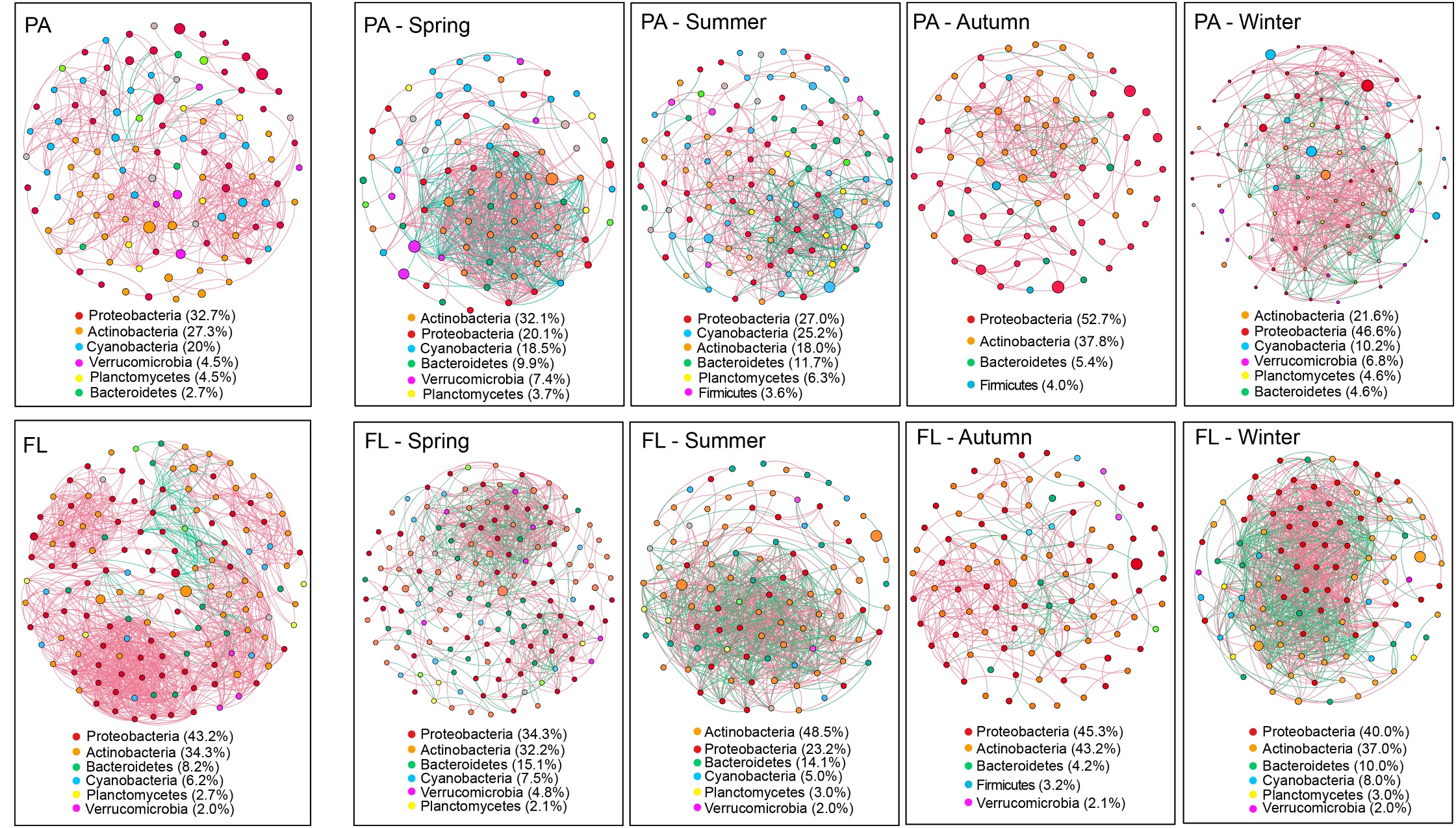


**Figure S7.** Correlation-based networks of abundant and frequent OTUs (relative abundance > 0.08%) in the PA and FL lifestyles. The size of each node is proportional to the number of connections (i.e., degree), and the nodes are colored according to different phyla. Numbers inside parentheses following names of each phylum represent relative proportion of nodes belonging to the phylum. Red and green edges indicate positive and negative correlations.
